# Antidepressant and Cognitive-Enhancing Effects of *Stewartia pseudocamellia* Maxim. Leaves in Chronic Unpredictable Mild Stress-Induced Mice Through HPA Axis Regulation and the BDNF/TrkB Pathway

**DOI:** 10.3390/ph19030354

**Published:** 2026-02-25

**Authors:** Yu Mi Heo, Hyo Lim Lee, Hye Ji Choi, Yeong Hyeon Ju, Hwa Rang Na, Ho Jin Heo

**Affiliations:** Division of Applied Life Science (BK21), Institute of Agriculture and Life Science, Gyeongsang National University, Jinju 52828, Republic of Korea; yumi@gnu.ac.kr (Y.M.H.); gyfla059@gnu.ac.kr (H.L.L.); hjchoi0820@gnu.ac.kr (H.J.C.); ju8172001@gnu.ac.kr (Y.H.J.); hrna@gnu.ac.kr (H.R.N.)

**Keywords:** *Stewartia pseudocamellia*, chronic unpredictable mild stress, antidepressant, cognitive function, HPA axis, inflammation, synaptic plasticity

## Abstract

**Background/Objectives**: *Stewartia pseudocamellia* Maxim. (*S. pseudocamellia*) has been reported to possess antioxidant and anti-inflammatory properties and contains various bioactive flavonoids and phenolic compounds. These components may contribute to neuroprotective effects relevant to depression and cognitive dysfunction. This study was conducted to evaluate the effects of 20% ethanolic extract from *S. pseudocamellia* leaves (ESP) on chronic unpredictable mild stress (CUMS)-induced depressive-like behaviors and cognitive dysfunction in C57BL/6 mice. **Methods**: C57BL/6 mice were divided into six groups: normal control (NC), normal sample (NS; ESP 100 mg/kg), CUMS, L-theanine (Thea; 4 mg/kg), ESP 50 mg/kg, and ESP 100 mg/kg groups. Phytochemical profiling of ESP was performed using ultra-performance liquid chromatography–quadrupole time-of-flight tandem mass spectrometry (UPLC-Q-TOF-MS/MS). Depressive-like behaviors and cognitive function were assessed, along with stress-related hormonal regulation and associated cellular signaling pathways. **Results**: Phytochemical profiling of ESP identified procyanidin B2, epicatechin, rutin, catechin gallate, kaempferol 3-O-glucoside, and quercitrin as major constituents. ESP significantly alleviated CUMS-induced depressive-like behaviors and improved spatial learning and memory. These effects were associated with modulation of stress-related hormones in serum and hypothalamic–pituitary–adrenal (HPA) axis–related proteins in the brain. ESP also enhanced antioxidant defense by activating the Nrf2 signaling pathway and improving mitochondrial function. Furthermore, ESP attenuated neuroinflammation and apoptosis by regulating the TLR4/NF-κB and JNK pathways, and promoted neuroplasticity by modulating cholinergic activity, with enhanced BDNF/TrkB signaling in the cerebral cortex and hippocampus. **Conclusions**: Collectively, these findings suggest that ESP exerts protective effects against CUMS-induced depressive-like behaviors and cognitive deficits in a preclinical model.

## 1. Introduction

Stress is an unavoidable aspect of daily life, a physiological and psychological response to external stimuli, and a crucial defense mechanism for maintaining body homeostasis [1]. While the body can adapt to acute stress, chronic stress disrupts nervous, immune, and endocrine functions, increasing the risk of severe mental disorders such as anxiety and major depressive disorder (MDD) [2]. MDD is accompanied by a range of symptoms, including persistent depression, loss of interest, and cognitive impairment [3]. It is strongly linked to a low quality of life and a high risk of suicide [3]. The World Health Organization (WHO) reports that more than 300 million people worldwide suffer from depression, making it a major mental health disorder with a significant social and economic burden [4]. Despite the high prevalence rates and severity, current standard treatments, such as antidepressants and psychotherapy, primarily focus on regulating monoamine neurotransmitters and fail to adequately address the complex etiology of depression [4,5]. In addition, the initiation of therapeutic effects necessitates an extended duration, and the occurrence of side effects such as anxiety, sleep disturbances, and cognitive impairments often limits their use [5,6]. Due to these limitations, there is growing interest in exploring safer and potentially beneficial complementary strategies [6].

Although the precise pathogenesis of depression has not yet been fully elucidated, several biological factors are known to be involved, such as the dysregulation of the hypothalamic-pituitary-adrenal (HPA) axis, neuroinflammation, oxidative stress, and monoamine deficiency [3,7]. Of these, chronic stress-induced hyperactivity of the HPA axis is considered a central pathological mechanism underlying depression [8]. Stress activates the HPA axis to induce the production of corticotropin-releasing factor (CRF) in the hypothalamus [7]. The increased levels of CRF induce the secretion of adrenocorticotropic hormone (ACTH) from the pituitary gland, which ultimately leads to elevated levels of glucocorticoids, including cortisol, from the adrenal gland [7]. Under normal conditions, glucocorticoids bind to the glucocorticoid receptor (GR) to maintain homeostasis of the HPA axis while modulating stress and immune responses [1]. However, chronic stress causes excessive activation of the HPA axis and a persistent increase in glucocorticoid levels, which in turn lead to impaired GR function and the overproduction of inflammatory cytokines, such as interleukin-1β (IL-1β) and tumor necrosis factor-α (TNF-α) [7,9,10]. These immune responses induce dysregulation of monoamine neurotransmitters, including serotonin, dopamine, and norepinephrine, which lead to behavioral abnormalities, such as depression, lethargy, and anxiety [10]. Chronic stress also leads to an overproduction of reactive oxygen species (ROS) that mediate mitochondrial dysfunction and neuronal damage, leading to overall cognitive impairments in memory and learning [11,12].

Cognitive impairments are closely associated with molecular changes at the synaptic level, including the suppression of the brain-derived neurotrophic factor (BDNF)/tropomyosin receptor kinase B (TrkB) signaling pathway and dysfunction of the cholinergic neurotransmission system, which are recognized as key pathophysiological mechanisms of stress-induced cognitive deficits [13,14]. Dysregulation of these pathways compromises synaptic structural integrity and reduces the efficiency of neural signal transmission, leading to various cognitive dysfunctions and depression [11,14]. Such neurobiological changes induced by chronic stress negatively impact both emotional and cognitive functions, emphasizing the need for intervention strategies to alleviate these pathophysiological changes. Recently, plant-based materials capable of positively modulating these neurobiological mechanisms have gained considerable attention as promising alternatives [8].

*Stewartia pseudocamellia* Maxim. (*S. pseudocamellia*) is a deciduous tree from the Theaceae family, distributed in Japan, China, and Korea [15]. In Korea, *S. pseudocamellia* is mainly native to the Jirisan region [15]. In traditional medicine, the bark and root bark of *S. pseudocamellia* have been used to alleviate blood stasis caused by contusions and to treat limb paralysis and neuralgia [16]. These pharmacological activities are attributed to various flavonoids and phenolic compounds as well as spinasterol glycoside, quercitrin, hyperin, and rutin, which are present in *S. pseudocamellia* [15,17]. Recent research has indicated that these constituents contribute to the antioxidant, anti-inflammatory, antimicrobial, and angiogenesis-promoting properties of *S. pseudocamellia* [17]. In a previous study, an ethanolic extract of *S. pseudocamellia* leaves was reported to attenuate H_2_O_2_-induced oxidative stress and neuronal cell damage in neuronal cells [18]. Moreover, our preliminary in vitro experiments showed that *S. pseudocamellia* leaves mitigated corticosterone-induced cytotoxicity and oxidative stress in neuronal cells, supporting its potential neuroprotective effects (Figure S1). However, whether these protective effects also extend to antidepressant and cognitive-enhancing outcomes under chronic stress conditions remains unclear, thereby warranting further in vivo investigation. Therefore, in this study, we aimed to investigate whether *S. pseudocamellia* leaves alleviate depressive-like behaviors and cognitive impairments using a mouse model of depression induced by chronic unpredictable mild stress (CUMS).

## 2. Results

### 2.1. Identification of Bioactive Compounds

**Figure 1:**
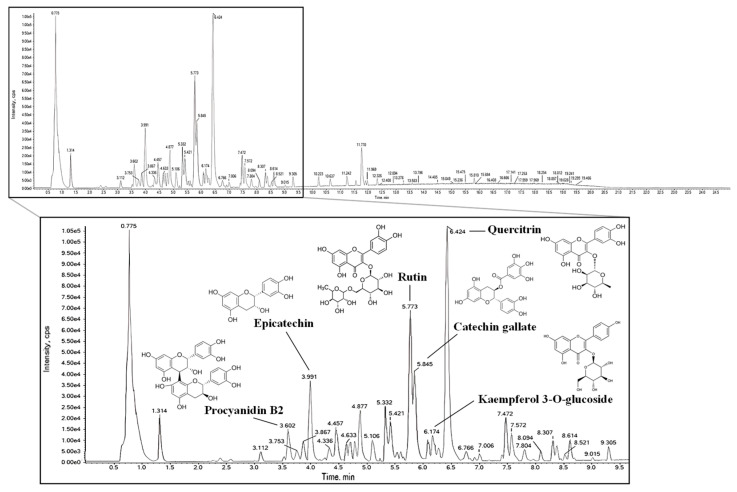
Ultra-performance liquid chromatography-quadrupole time-of-flight tandem mass spectrometry (UPLC-Q-TOF-MS/MS) chromatogram of 20% ethanolic extract from *Stewartia pseudocamellia* Maxim. leaves (ESP).

The bioactive compounds in 20% ethanolic extract from *S. pseudocamellia* leaves (ESP) were identified using ultra-performance liquid chromatography-quadrupole time-of-flight tandem mass spectrometry (UPLC-Q-TOF-MS/MS), and the results are presented in Figure 1 and Table 1. The MS spectra revealed the presence of the following compounds: procyanidin B2 (RT, 3.602 min; parent ion, 577; fragment ions, 425, 407, and 289), epicatechin (RT, 3.991 min; parent ion, 579; fragment ions, 289 and 245), rutin (RT, 5.773 min; parent ion, 609; fragment ions, 301, 300, 271, 255, and 151), catechin gallate (RT, 5.845 min; parent ion, 441; fragment ions, 289, 169, and 125), kaempferol 3-O-glucoside (RT, 6.174 min; parent ion, 447; fragment ions, 285, 284, 255 and 227), and quercitrin (RT, 6.424 min; parent ion, 447; fragment ions, 301, 300, and 151).

### 2.2. Quantitative Analysis of Bioactive Compounds

**Figure 2:**
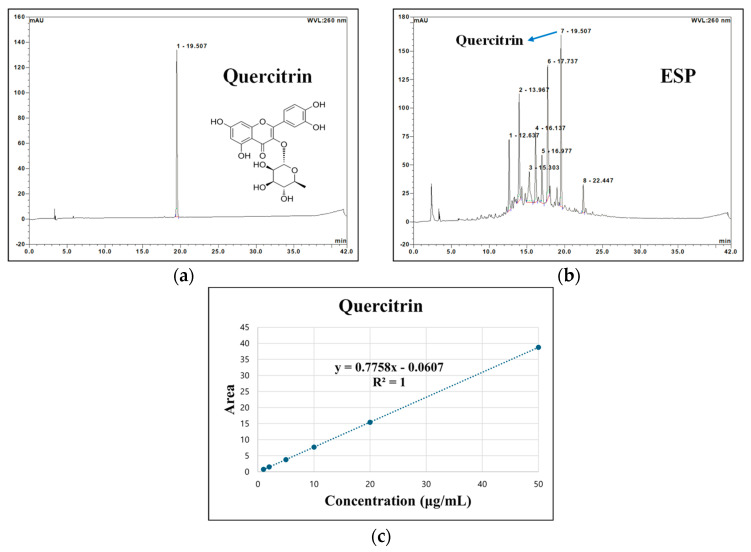
High-performance liquid chromatography–diode array detector (HPLC-DAD) chromatograms of the quercitrin standard (**a**) and 20% ethanolic extract from *Stewartia pseudocamellia* Maxim. Leaves (ESP) (**b**) acquired at 260 nm. Calibration curve for quercitrin quantitation (**c**).

Representative high-performance liquid chromatography–diode array detector (HPLC-DAD) chromatograms of the quercitrin standard and ESP acquired at 260 nm are presented in Figure 2. Quercitrin was detected in the extract at the same retention time as the authentic standard. A calibration curve was constructed from the linear relationship between peak area and concentration of the standard solution over the range of 1–50 μg/mL, demonstrating excellent linearity. Using this calibration curve, the concentration of quercitrin in ESP was determined to be 23.00 ± 0.01 μg/mg of dry weight. In addition, the rutin content of the extract was previously determined in our earlier study and was 21.40 ± 0.09 μg/mg of dry weight [18].

### 2.3. Effect of ESP on Behavioral Tests

**Figure 3:**
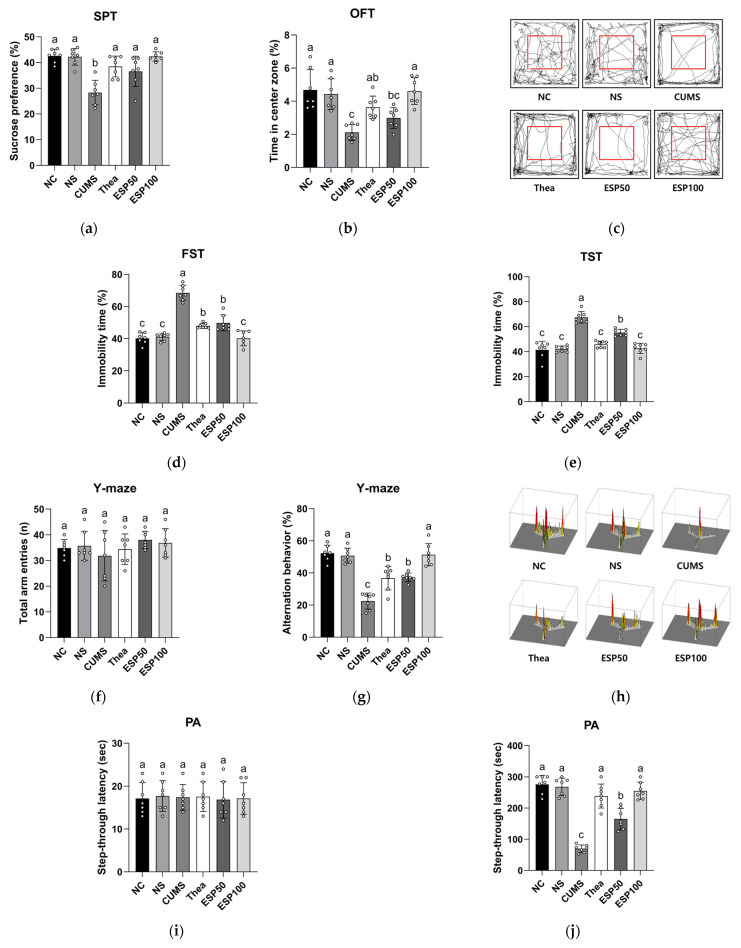

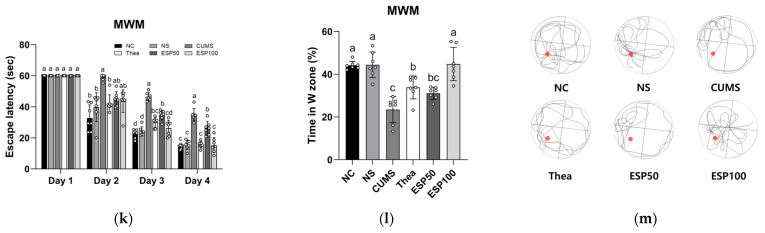
Effect of 20% ethanolic extract from *Stewartia pseudocamellia* Maxim. Leaves (ESP) on behavioral tests in CUMS-induced mice. Sucrose preference in sucrose preference test (SPT) (**a**), time in center zone (**b**) and path tracing of respective group (**c**) in open field test (OFT), immobility time in forced swim test (FST) (**d**), immobility time in tail suspension test (TST) (**e**), total arm entries (**f**), alternation behavior (**g**), and 3D image of path tracing (**h**) in the Y-maze test, step-through latency during habituation phase (**i**) and test phase (**j**) in passive avoidance (PA) test, escape latency during hidden test (**k**), time in W zone during probe test (**l**), and swimming pattern visualization image during probe test (**m**) in the Morris water maze (MWM) test. Values are presented as the mean ± SD (*n* = 7). Different lowercase letters (a–d) above the bars indicate statistically significant differences between groups (*p* < 0.05). Bars marked with the same letter indicate no statistically significant differences between groups. One-way ANOVA followed by Tukey’s post hoc test was applied for statistical analysis, except for escape latency during hidden test (**k**) in the MWM test, which was analyzed using the Kruskal–Wallis test followed by Dunn’s post hoc test.

Previous studies have reported that theanine alleviates stress-induced behavioral impairments and exerts antidepressant-like and cognition-enhancing effects through the regulation of neurotransmitter systems and neuroplasticity [19]. On this basis, theanine was utilized as a positive control in this study. The results of evaluating the effect of ESP on depressive-like behaviors and cognitive impairments in CUMS-induced mice are presented in Figure 3.

In the sucrose preference test (SPT), the CUMS group exhibited reduced sucrose preference relative to the normal control (NC) group (*p* < 0.001). However, the L-theanine (Thea) group demonstrated a significant increase in sucrose preference relative to the CUMS group (*p* < 0.001). In addition, treatment with ESP showed a substantial enhancement in sucrose preference relative to the CUMS group (*p* < 0.001) (Figure 3a).

In the open field test (OFT), the CUMS group showed decreased time spent in the center zone relative to the NC group (*p* < 0.001). In contrast, the Thea group exhibited a significant increase in time spent in the center zone relative to the CUMS group (*p* < 0.05). Likewise, treatment with ESP demonstrated a marked improvement in time spent in the center zone relative to the CUMS group (*p* < 0.001) (Figure 3b). In addition, the path-tracing analysis showed that the CUMS group predominantly remained within the peripheral zone relative to other groups (Figure 3c).

In the forced swim test (FST), the CUMS group exhibited increased immobility time relative to the NC group (*p* < 0.001). However, the Thea group showed a significant reduction in immobility time relative to the CUMS group (*p* < 0.001). Moreover, treatment with ESP resulted in a substantial decrease in immobility time relative to the CUMS group (*p* < 0.001) (Figure 3d).

In the tail suspension test (TST), the CUMS group displayed elevated immobility time relative to the NC group (*p* < 0.001). In contrast, the Thea group demonstrated a significant decrease in immobility time relative to the CUMS group (*p* < 0.001). Similarly, treatment with ESP showed a marked reduction in immobility time relative to the CUMS group (*p* < 0.001) (Figure 3e).

In the Y-maze test, no significant differences were observed in the number of total arm entries of the mice among the groups (Figure 3f). The CUMS group exhibited reduced alternation behavior relative to the NC group (*p* < 0.001). Conversely, the Thea group showed a significant improvement in alternation behavior relative to the CUMS group (*p* < 0.005). Furthermore, treatment with ESP resulted in a substantial increase in alternation behavior relative to the CUMS group (*p* < 0.001) (Figure 3g). Additionally, 3D path tracing results indicated that the CUMS group tended to enter a specific arm more frequently than the other groups (Figure 3h).

In the passive avoidance (PA) test, no significant differences were noted in the step-through latency among the groups during the habituation phase on the first day (Figure 3i). However, during the test phase on the second day, the CUMS group displayed reduced step-through latency relative to the NC group (*p* < 0.001). In contrast, the Thea group showed a significant extension of step-through latency relative to the CUMS group (*p* < 0.001). Likewise, treatment with ESP exhibited a substantial prolongation of step-through latency relative to the CUMS group (*p* < 0.001) (Figure 3j).

In the Morris water maze (MWM) test, no significant differences were observed in escape latency among the groups on the first day (Figure 3k). However, following four days of training, the CUMS group exhibited increased escape latency relative to the NC group (*p* < 0.001). However, the Thea group showed a significant reduction in escape latency relative to the CUMS group (*p* < 0.001). Similarly, treatment with ESP markedly shortened the escape latency relative to the CUMS group (*p* < 0.001). In the probe test, the CUMS group displayed reduced time spent in the W zone relative to the NC group (*p* < 0.001). Conversely, the Thea group demonstrated a significant prolongation of time spent in the W zone relative to the CUMS group (*p* < 0.05). Furthermore, treatment with ESP markedly enhanced time spent in the W zone relative to the CUMS group (*p* < 0.001) (Figure 3l). Moreover, path tracing results revealed that the CUMS group spent less time in the W zone than the other groups (Figure 3m).

### 2.4. Effect of ESP on Stress-Related Hormone

The results of measuring the effect of ESP on serum levels of stress-related hormones in CUMS-induced mice are presented in Table 2. The CUMS group exhibited elevated corticosterone levels relative to the NC group (*p* < 0.001). However, the ESP 100 treatment resulted in a statistically significant reduction in corticosterone levels relative to the CUMS group (*p* < 0.001). The CUMS group showed decreased dopamine and serotonin levels relative to the NC group (*p* < 0.01 or less). In contrast, the ESP 100 treatment exhibited a significant increase in dopamine and serotonin levels relative to the CUMS group (*p* < 0.05 or less).

### 2.5. Effect of ESP on HPA Axis-Related Pathway

**Figure 4:**
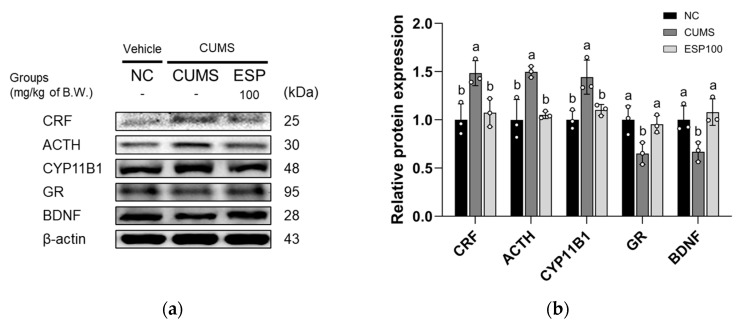
Effect of 20% ethanolic extract from *Stewartia pseudocamellia* Maxim. Leaves (ESP) on hypothalamic-pituitary-adrenal (HPA) axis-related pathway of brain tissue in CUMS-induced mice. Western blot images of protein bands (**a**). Relative protein expression of CRF, ACTH, CYP11B1, GR, and BDNF (**b**) was normalized to β-actin. Values are presented as the mean ± SD (*n* = 3). Different lowercase letters (a, b) above the bars indicate statistically significant differences between groups (*p* < 0.05). Bars marked with the same letter indicate no statistically significant differences between groups. One-way ANOVA followed by Tukey’s post hoc test was applied for statistical analysis.

The results of assessing the anti-stress effect of ESP on the expression levels of HPA axis-related proteins in CUMS-induced mice are presented in Figure 4. The CUMS group exhibited upregulated expression of CRF, ACTH, and cytochrome P450 family 11 subfamily B member 1 (CYP11B1) relative to the NC group (*p* < 0.05 or less). However, the ESP 100 group displayed a significant downregulation of CRF, ACTH, and CYP11B1 expression relative to the CUMS group (*p* < 0.05). The CUMS group showed reduced expression of GR and BDNF relative to the NC group (*p* < 0.05). Conversely, the ESP 100 group demonstrated a significant increase in GR and BDNF expression relative to the CUMS group (*p* < 0.05).

### 2.6. Effect of ESP on Antioxidant System

**Figure 5:**
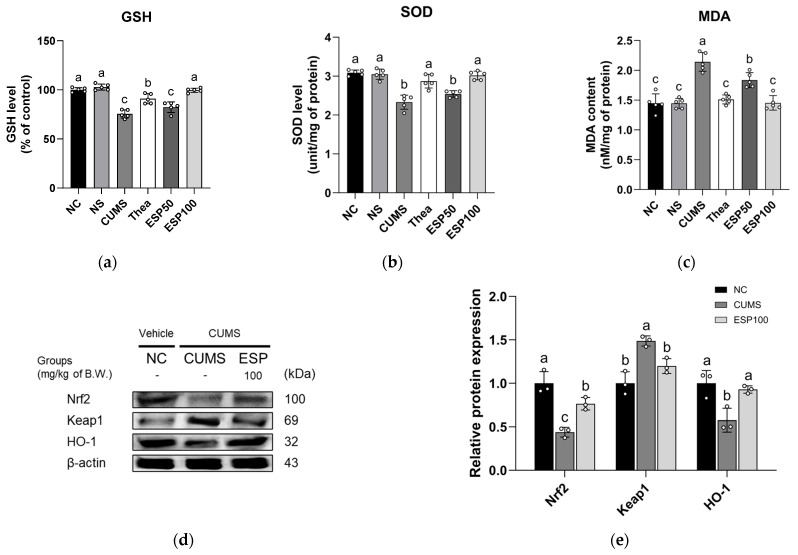
Effect of 20% ethanolic extract from *Stewartia pseudocamellia* Maxim. Leaves (ESP) on antioxidant system of brain tissue in CUMS-induced mice. Reduced glutathione (GSH) level (**a**), superoxide dismutase (SOD) level (**b**), and malondialdehyde (MDA) content (**c**) (*n* = 5). Western blot images of protein bands (**d**). Relative protein expression of Nrf2, Keap1, and HO-1 (**e**) was normalized to β-actin (*n* = 3). Values are presented as the mean ± SD. Different lowercase letters (a–c) above the bars indicate statistically significant differences between groups (*p* < 0.05). Bars marked with the same letter indicate no statistically significant differences between groups. One-way ANOVA followed by Tukey’s post hoc test was applied for statistical analysis.

The results of examining the antioxidative effect of ESP on oxidative stress markers and the expression levels of nuclear factor erythroid 2-related factor 2 (Nrf2) signaling pathway proteins in CUMS-induced mice are presented in Figure 5.

The CUMS group exhibited decreased reduced glutathione (GSH) level relative to the NC group (*p* < 0.001). However, the Thea group showed a significant increase in GSH level relative to the CUMS group (*p* < 0.001). Furthermore, treatment with ESP resulted in a marked elevation of GSH level relative to the CUMS group (*p* < 0.001) (Figure 5a).

The CUMS group displayed a decline in superoxide dismutase (SOD) level relative to the NC group (*p* < 0.001). Conversely, the Thea group demonstrated a significant enhancement of SOD level relative to the CUMS group (*p* < 0.001). Likewise, treatment with ESP led to a pronounced improvement in SOD levels relative to the CUMS group (*p* < 0.001) (Figure 5b).

The CUMS group presented elevated malondialdehyde (MDA) content relative to the NC group (*p* < 0.001). In contrast, the Thea group displayed a significant decline in MDA content relative to the CUMS group (*p* < 0.001). Similarly, treatment with ESP resulted in a marked attenuation of MDA content relative to the CUMS group (*p* < 0.001) (Figure 5c).

The CUMS group exhibited downregulated expression of Nrf2 and heme oxygenase 1 (HO-1) relative to the NC group (*p* < 0.05 or less). However, the ESP 100 group displayed a significant upregulation of Nrf2 and HO-1 expression relative to the CUMS group (*p* < 0.05). The CUMS group showed elevated expression of Kelch-like ECH-associated protein 1 (Keap1) relative to the NC group (*p* < 0.005). In contrast, the ESP 100 group demonstrated a significant reduction in Keap1 expression relative to the CUMS group (*p* < 0.05) (Figure 5d,e).

### 2.7. Effect of ESP on Neuroinflammation

**Figure 6:**
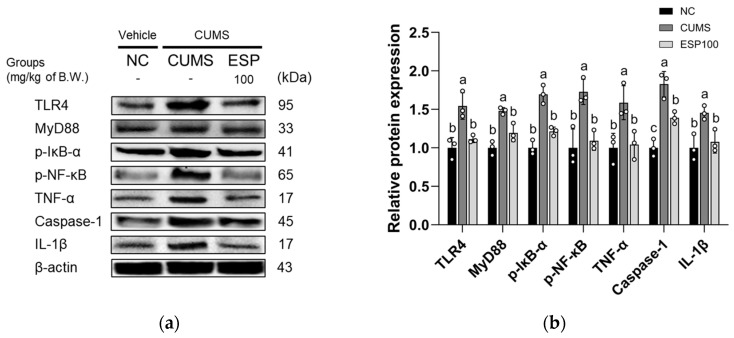
Effect of 20% ethanolic extract from *Stewartia pseudocamellia* Maxim. Leaves (ESP) on neuroinflammation of brain tissue in CUMS-induced mice. Western blot images of protein bands (**a**). Relative protein expression of TLR4, MyD88, p-IκB-α, p-NF-κB, TNF-α, caspase-1, and IL-1β (**b**) was normalized to β-actin. Values are presented as the mean ± SD (*n* = 3). Different lowercase letters (a–c) above the bars indicate statistically significant differences between groups (*p* < 0.05). Bars marked with the same letter indicate no statistically significant differences between groups. One-way ANOVA followed by Tukey’s post hoc test was applied for statistical analysis.

The results of evaluating the anti-inflammatory effect of ESP on the expression levels of Toll-like receptor 4 (TLR4) signaling pathway proteins in CUMS-induced mice are shown in Figure 6. The CUMS group showed elevated expression of TLR4, myeloid differentiation primary response 88 (MyD88), phosphorylated inhibitor of κBα (p-IκB-α), phosphorylated nuclear factor-κB (p-NF-κB), TNF-α, caspase-1, and IL-1β relative to the NC group (*p* < 0.05 or less). However, the ESP 100 group exhibited a significant reduction in TLR4, MyD88, p-IκB-α, p-NF-κB, TNF-α, caspase-1, and IL-1β expression relative to the CUMS group (*p* < 0.05 or less).

### 2.8. Effect of ESP on Mitochondrial Dysfunction and Neurotoxicity

**Figure 7:**
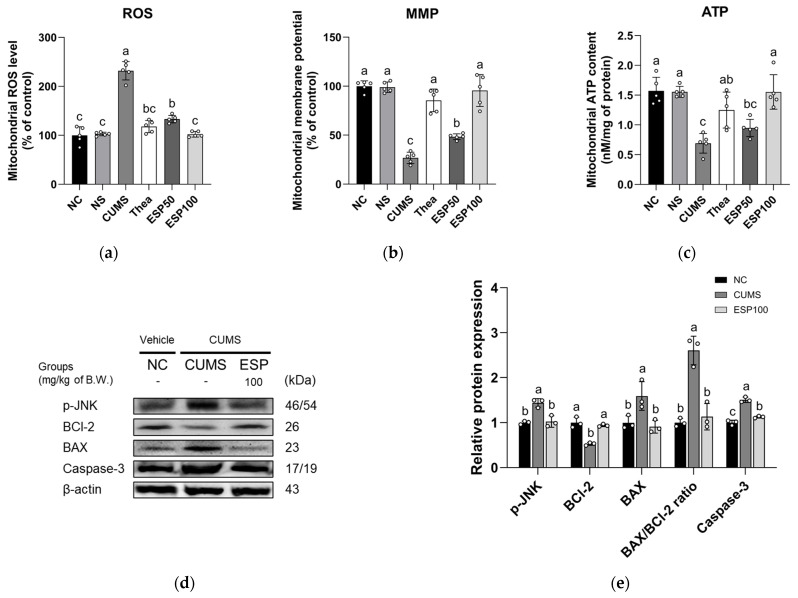
Effect of 20% ethanolic extract from *Stewartia pseudocamellia* Maxim. Leaves (ESP) on mitochondrial function and neurotoxicity of brain tissue in CUMS-induced mice. Mitochondrial reactive oxygen species (ROS) level (**a**), mitochondrial membrane potential (MMP) (**b**), and mitochondrial adenosine triphosphate (ATP) content (**c**) (*n* = 5). Western blot images of protein bands (**d**). Relative protein expression of p-JNK, BCl-2, BAX, BAX/BCl-2 ratio, and caspase-3 (**e**) was normalized to β-actin (*n* = 3). Values are presented as the mean ± SD. Different lowercase letters (a–c) above the bars indicate statistically significant differences between groups (*p* < 0.05). Bars marked with the same letter indicate no statistically significant differences between groups. One-way ANOVA followed by Tukey’s post hoc test was applied for statistical analysis.

The results of investigating the neuroprotective effect of ESP on mitochondrial dysfunction and the expression levels of c-Jun N-terminal kinase (JNK) signaling pathway proteins in CUMS-induced mice are presented in Figure 7.

The CUMS group showed elevated mitochondrial ROS level relative to the NC group (*p* < 0.001). In contrast, the Thea group exhibited a significant reduction in mitochondrial ROS level relative to the CUMS group (*p* < 0.001). Moreover, treatment with ESP led to a marked suppression of mitochondrial ROS level relative to the CUMS group (*p* < 0.001) (Figure 7a).

The CUMS group exhibited a decline in mitochondrial membrane potential (MMP) relative to the NC group (*p* < 0.001). However, the Thea group demonstrated a significant restoration of MMP relative to the CUMS group (*p* < 0.001). Likewise, treatment with ESP resulted in a marked improvement of MMP relative to the CUMS group (*p* < 0.001) (Figure 7b).

The CUMS group showed decreased mitochondrial adenosine triphosphate (ATP) content relative to the NC group (*p* < 0.001). Conversely, the Thea group displayed a significant enhancement of mitochondrial ATP content relative to the CUMS group (*p* < 0.01). Furthermore, treatment with ESP led to a notable restoration of mitochondrial ATP content relative to the CUMS group (*p* < 0.001) (Figure 7c).

The CUMS group showed increased expression of p-JNK, BCl-2 associated X (BAX), BAX/B-cell leukemia/lymphoma 2 (BCl-2) ratio, and caspase-3 relative to the NC group (*p* < 0.005). In contrast, the ESP 100 group demonstrated a significant reduction in p-JNK, BAX, BAX/BCl-2 ratio, and caspase-3 expression relative to the CUMS group (*p* < 0.005). The CUMS group exhibited decreased expression of BCl-2 relative to the NC group (*p* < 0.05). However, the ESP 100 group displayed a significant enhancement of BCl-2 expression relative to the CUMS group (*p* < 0.05) (Figure 7d,e).

### 2.9. Effect of ESP on Neuroplasticity

**Figure 8:**
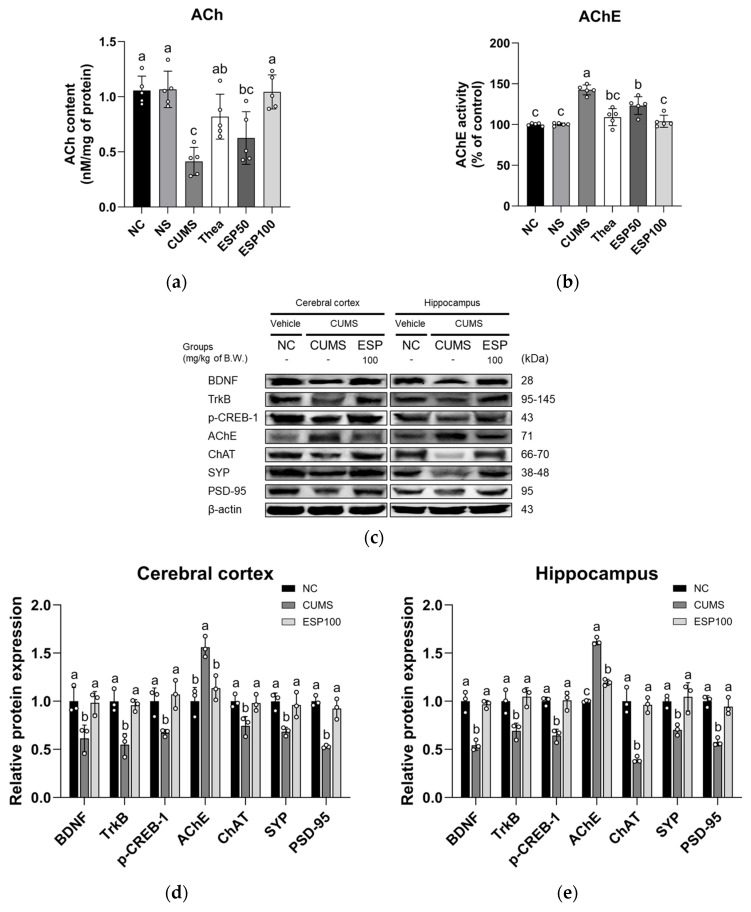
Effect of 20% ethanolic extract from *Stewartia pseudocamellia* Maxim. leaves (ESP) on neuroplasticity of brain tissue in CUMS-induced mice. Acetylcholine (ACh) content (**a**) and acetylcholinesterase (AChE) activity (**b**) in whole brain (*n* = 5). Western blot images of protein bands (**c**). Relative protein expression of BDNF, TrkB, p-CREB-1, AChE, ChAT, SYP, and PSD-95 in cerebral cortex (**d**) and hippocampus (**e**) was normalized to β-actin (*n* = 3). Values are presented as the mean ± SD. Different lowercase letters (a–c) above the bars indicate statistically significant differences between groups (*p* < 0.05). Bars marked with the same letter indicate no statistically significant differences between groups. One-way ANOVA followed by Tukey’s post hoc test was applied for statistical analysis.

The results of assessing the neuroplasticity-enhancing effect of ESP on the cholinergic system and the expression levels of cholinergic enzymes and synaptic plasticity-related proteins in CUMS-induced mice are presented in Figure 8.

The CUMS group exhibited reduced acetylcholine (ACh) content relative to the NC group (*p* < 0.001). However, the Thea group showed a significant restoration of ACh content relative to the CUMS group (*p* < 0.05). Moreover, treatment with ESP showed a substantial increase in ACh content relative to the CUMS group (*p* < 0.001) (Figure 8a).

The CUMS group exhibited increased acetylcholinesterase (AChE) activity relative to the NC group (*p* < 0.001). In contrast, the Thea group demonstrated a significant decrease in AChE activity relative to the CUMS group (*p* < 0.001). Likewise, treatment with ESP showed a marked suppression of AChE activity relative to the CUMS group (*p* < 0.001) (Figure 8b).

The CUMS group exhibited elevated expression of AChE in the cerebral cortex and hippocampus relative to the NC group (*p* < 0.005). However, the ESP 100 group showed a statistically significant reduction in AChE expression in the cerebral cortex and hippocampus relative to the CUMS group (*p* < 0.05). The CUMS group displayed decreased expression of BDNF, TrkB, phosphorylated cAMP response element-binding protein 1 (p-CREB-1), choline acetyltransferase (ChAT), synaptophysin (SYP), and postsynaptic density protein 95 (PSD-95) in the cerebral cortex and hippocampus relative to the NC group (*p* < 0.05 or less). Conversely, the ESP 100 group exhibited a statistically significant increase in BDNF, TrkB, p-CREB-1, ChAT, SYP, and PSD-95 expression in the cerebral cortex and hippocampus relative to the CUMS group (*p* < 0.05 or less) (Figure 8c–e).

### 2.10. Correlation Between Depression-Related and Cognitive Dysfunction Biomarkers

**Figure 9:**
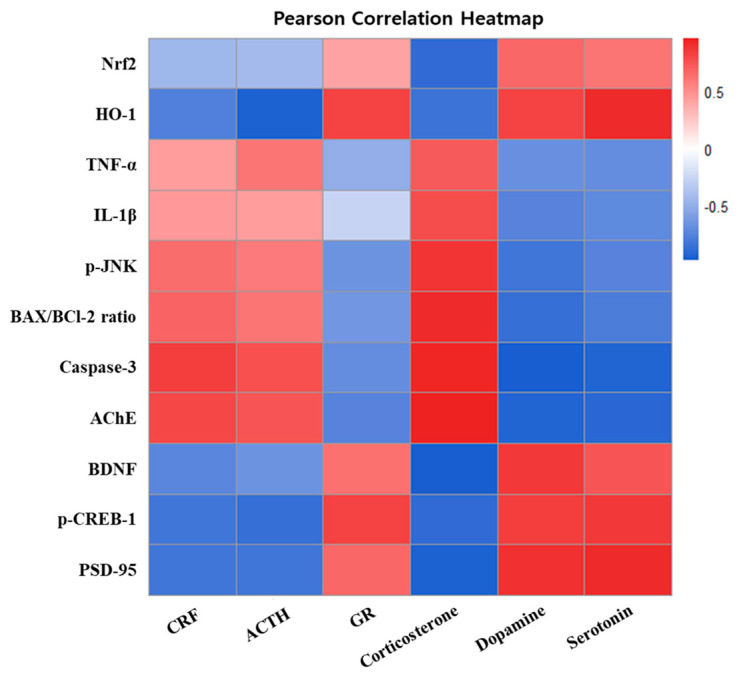
Pearson correlation heatmap between depression-related and cognitive dysfunction biomarkers in CUMS-induced mice. Color encodes Pearson correlations (red = positive, blue = negative), and intensity reflects correlation strength.

The results of the Pearson correlation analysis between depression-related and cognitive dysfunction biomarkers in CUMS-induced mice are presented in Figure 9. As key mediators of the HPA axis, CRF, ACTH, and corticosterone exhibited positive correlations with inflammatory and apoptotic factors, including TNF-α, IL-1β, p-JNK, the BAX/BCl-2 ratio, and caspase-3, as well as with the cholinergic enzyme AChE. In contrast, GR demonstrated an opposite correlation pattern. It showed positive correlations with the antioxidant markers Nrf2 and HO-1 and with synaptic plasticity-related proteins such as BDNF, p-CREB-1, and PSD-95, while exhibiting negative correlations with inflammatory and apoptotic factors. Similarly, the monoaminergic neurotransmitters dopamine and serotonin exhibited a comparable correlation pattern to GR.

## 3. Discussion

Chronic stress, one of the significant risk factors for depression, has been shown to adversely affect emotional regulation and cognitive functions and is associated with dysregulation of the HPA axis, neuroinflammatory responses, and synaptic dysfunction [20,21,22]. These mechanisms have been widely implicated in depressive symptoms and stress-related cognitive decline, underscoring the need for effective strategies to restore neural homeostasis [10,21]. In this context, plant-derived bioactive compounds are increasingly highlighted as promising candidates because they exert antioxidative, anti-inflammatory, and neuroprotective effects, and possess the capacity to modulate stress-related pathways [5]. Therefore, the present study investigated the protective effects of ESP on depressive-like behavioral and cognitive dysfunction induced by CUMS.

Since the biological activity of natural products is closely associated with their chemical composition, we performed UPLC-Q-TOF-MS/MS to analyze the constituents of ESP. The analysis confirmed that ESP contains multiple phenolic compounds, including procyanidin B2, epicatechin, rutin, catechin gallate, kaempferol 3-O-glucoside, and quercitrin (Figure 1 and Table 1). These phenolic compounds are widely recognized for their potent antioxidant, anti-inflammatory, and neuroprotective properties [5,23,24]. In particular, quercitrin, a major compound of ESP, exhibited anti-inflammatory and neuroprotective effects in animal models of emotional and cognitive dysfunction, while rutin provided neuroprotection by reducing oxidative stress and preventing hippocampal damage [25,26]. Similarly to previous findings, our preliminary in vitro experiments revealed the neuroprotective potential of ESP as it reduced cytotoxicity and oxidative stress in corticosterone-induced HT22 mouse hippocampal neuronal cells and MC-IXC human fibroblast cells (Figure S1). However, these cellular responses do not necessarily reflect behavioral and cognitive dysfunction observed in vivo. Therefore, we further evaluated the protective effects of ESP in a CUMS-induced mouse model of emotional and cognitive dysfunction.

The CUMS model is a widely employed and validated preclinical paradigm for depression, as it reproduces the chronic and unpredictable nature of human psychosocial stress and induces persistent depression-like phenotypes in rodents, including anhedonia, reduced exploratory activity, and behavioral despair [19]. Beyond these behavioral changes, CUMS is associated with widespread neurobiological alterations, including dysregulation of the HPA axis and monoaminergic signaling, as well as increased oxidative stress and neuroinflammatory activity [27,28,29,30,31]. These pathophysiological alterations converge to impair emotional regulation and reward processing, providing a mechanistic basis for the depression-like behaviors observed in the CUMS model [27,28]. Accordingly, to assess stress-induced behavioral disturbances in a preclinical model, CUMS models commonly utilize behavioral tests such as the SPT, OFT, FST, and TST [27]. These tests reflect core depressive symptoms and are widely used to assess emotional dysfunction [5,27]. On this basis, we investigated whether ESP could ameliorate depression-related behavioral impairments in CUMS-induced mice. The results demonstrated that CUMS exposure led to significant behavioral deficits in the SPT, OFT, FST, and TST, whereas ESP administration markedly ameliorated these abnormalities (Figure 3a–e). According to a previous study, rutin, a primary bioactive compound in ESP, improved behavioral abnormalities in the OFT, FST, and TST by suppressing HPA axis hyperactivation, thereby normalizing corticosterone levels, and by inhibiting monoamine oxidase A (MAO-A) activity, leading to increased monoamine availability [5]. Another study demonstrated that quercitrin markedly reduced immobility time in both the FST and TST, alleviating stress-induced depressive-like behaviors [25]. Similarly, aqueous extracts of *Camellia euphlebia*, which belongs to the same plant family (Theaceae) as ESP, significantly reduced immobility duration in both the forced swimming test and tail suspension test in mice [32]. Our findings suggest that the phenolic constituents of ESP, including rutin and quercitrin, may contribute to improvements in stress-induced depressive-like behaviors, potentially through modulation of HPA axis activity. Although the specific contribution of individual phenolic compounds was not directly examined in this study, these behavioral improvements are consistent with previously reported neuroendocrine regulatory actions of these constituents. Taken together, these results indicate that ESP exerts protective effects against chronic stress–induced emotional disturbances in a preclinical model.

Beyond emotional regulation, chronic stress impairs learning and memory, which has been associated with neuroinflammation and oxidative damage in stress-sensitive brain regions such as the hippocampus [33]. Repeated stress stimuli have been reported to activate the nucleotide-binding oligomerization domain (NOD)-like receptor family pyrin domain containing 3 (NLRP3) inflammasome and increase pro-inflammatory cytokine production, changes that have been associated with reduced BDNF expression and impaired neuroplasticity [33]. Additionally, prolonged corticosterone exposure further aggravates hippocampal structural and functional deficits, contributing to cognitive decline [28,34]. Consistent with these mechanisms, CUMS-induced mice in our study showed marked impairments in learning and memory in the Y-maze, PA, and MWM tests (Figure 3f–m). However, ESP administration significantly alleviated these deficits, leading to improvements in working memory and spatial learning. These protective effects could also be linked to the bioactive compounds in ESP. Kaempferol 3-O-glucoside has been reported to inhibit NLRP3 inflammasome activity, thereby reducing inflammatory cytokine secretion and mitigating cognitive impairments associated with neuroinflammation [35]. Moreover, quercitrin has been shown to enhance neuroplasticity by upregulating hippocampal BDNF and synaptic proteins [25]. Taken together, these results suggest that ESP exerts protective effects against CUMS-induced memory impairment and spatial learning deficits in a preclinical model, potentially through the complementary actions of its phenolic constituents. To further elucidate the biological pathways underlying these behavioral improvements, additional analyses were performed.

Chronic stress disrupts neuroendocrine homeostasis by altering the metabolism of key hormones and neurotransmitters [36,37]. Activation of the HPA axis under stress involves CRF-induced ACTH release, which promotes corticosterone synthesis via steroidogenic enzymes such as CYP11B1 [1,7,29,38]. Under normal conditions, secreted corticosterone acts on the hypothalamus and pituitary gland to maintain HPA axis homeostasis via a negative feedback mechanism that inhibits CRF and ACTH secretion [38]. However, prolonged stress disrupts this regulation, leading to sustained corticosterone elevation and reduced GR expression and sensitivity [21,29,34,38]. Such GR dysfunction has been associated with alterations in BDNF-dependent neuroplasticity that may contribute to synaptic impairment and depressive pathology [39]. Moreover, excessive corticosterone and HPA axis hyperactivation have been linked to monoaminergic disturbances [29]. In particular, chronic stress perturbs the tryptophan pathway by increasing indoleamine 2,3-dioxygenase (IDO) enzymatic activity and enhancing MAO-mediated serotonin degradation, while sustained corticosterone exposure impairs mesolimbic dopamine transporter function, reducing reward responsiveness and hedonic processing [40,41]. Collectively, these neuroendocrine and neurotransmitter alterations not only weaken stress adaptability but also exacerbate emotional dysregulation and depressive symptoms [8]. In alignment with these observations, our results demonstrated that CUMS induced an upregulation in the expression levels of CRF, ACTH, and CYP11B1, while downregulating GR and BDNF, reflecting hyperactivation of the HPA axis and impaired negative feedback regulation (Figure 4). These alterations were accompanied by altered serum levels of corticosterone, dopamine, and serotonin (Table 2). In contrast, ESP administration effectively attenuated these pathological changes. These protective effects may be associated with the phenolic compounds contained in ESP, including quercitrin and rutin. Both are glycosides of quercetin, a flavonol that is metabolized to quercetin upon ingestion [42]. Quercetin and its derivatives have been reported to alleviate HPA axis hyperactivation by suppressing corticosterone and CRF under stress conditions, thereby improving memory impairment and stress-related disorders [6]. In parallel, rutin restores HPA axis homeostasis by normalizing ACTH and corticosterone levels in CUMS-exposed animals [5]. Moreover, these compounds have been shown to restore the functional balance of stress-disrupted neuroendocrine and neurotransmitter systems through the normalization of monoaminergic metabolism [5,43]. In particular, serotonin restoration appears to result from stabilization of the tryptophan pathway and suppression of aberrant MAO activity, thereby enhancing serotonergic neurotransmission [40]. In line with these observations, extracts from another Theaceae family plant, *Camellia euphlebia*, were reported to reduce ACTH and corticosterone levels in CUMS-induced mice and to increase serotonin, noradrenaline, and dopamine levels [32]. Collectively, these results indicate that ESP ameliorates stress-induced disruptions of the HPA axis and monoaminergic systems, which is associated with improvements in neuroendocrine regulation and synaptic plasticity. These mechanistic improvements are consistent with the behavioral recovery observed in our assays and further support the modulatory effects of ESP on stress-induced neuroendocrine and monoaminergic dysregulation in a preclinical model.

Chronic stress is known to disrupt redox homeostasis in the brain, leading to excessive ROS accumulation and subsequent oxidative stress, thereby compromising neuronal structure and function [30]. Sustained oxidative stress has been reported to be associated with depletion of endogenous antioxidants such as GSH and SOD, thereby weakening the cellular antioxidant defense system [5,44,45]. Among the primary antioxidant defense mechanisms, the Nrf2/Keap1/HO-1 signaling pathway plays a pivotal role in maintaining redox balance by regulating the transcription of antioxidant enzymes [46]. Under physiological conditions, Nrf2 dissociates from Keap1 in response to oxidative stimuli, translocates to the nucleus, and induces the expression of antioxidant-related genes [44,46]. However, prolonged oxidative stress can reduce antioxidant capacity by disrupting this regulatory process [44,46]. Excessive ROS has been implicated in amplifying neuroinflammatory signaling, including increased production of pro-inflammatory cytokines via diverse inflammatory pathways [45]. In particular, CUMS-induced stress has been shown to activate the TLR4/MyD88 pathway, thereby promoting inflammatory responses [47]. This activation facilitates NF-κB nuclear translocation, leading to enhanced expression of inflammatory mediators, such as TNF-α and IL-1β, and exacerbating neuroinflammation [45,47]. These cytokines disrupt monoaminergic neurotransmission by reducing tryptophan availability and activating the IDO pathway, while also depleting tetrahydrobiopterin (BH4), an essential cofactor for dopamine synthesis, ultimately impairing both serotonergic and dopaminergic signaling [5,31]. Along with these monoaminergic disturbances, sustained neuroinflammation has been associated with neuronal injury and synaptic dysfunction, which may contribute to the cognitive impairment characteristic of depression [5,31]. Consistent with these mechanistic insights, our results demonstrate that CUMS induces oxidative stress by suppressing the Nrf2 antioxidant pathway while simultaneously enhancing inflammatory signaling through TLR4/NF-κB activation (Figure 5 and Figure 6). In contrast, administration of ESP significantly attenuated these stress-induced alterations, restoring antioxidant defense-related signaling and suppressing excessive inflammatory activation. In line with our observations, studies have reported that phenolic compounds such as epicatechin inhibit ROS production and ameliorate neuronal dysfunction by promoting Nrf2 nuclear translocation and enhancing antioxidant-related protein expression in brain tissues [24]. Furthermore, rutin has been shown to exert neuroprotective effects under Pb-induced oxidative stress conditions in SH-SY5Y cells by activating Nrf2-mediated HO-1 expression while concurrently reducing TNF-α and IL-1β levels [44]. Taken together, these findings suggest that ESP may mitigate chronic stress–induced neuronal dysfunction by modulating oxidative stress and neuroinflammatory pathways.

Sustained neuroinflammation induced by chronic stress has been reported to disrupt neuronal homeostasis and to be associated with mitochondrial dysfunction [10,48]. Mitochondria are the primary source of intracellular ATP and play a central role in maintaining calcium homeostasis and regulating apoptotic processes [48,49,50]. Persistent inflammation has been reported to exacerbate oxidative damage and to impair mitochondrial membrane potential and ATP synthesis [48]. Given that synaptic assembly and neurotransmission are highly dependent on mitochondrial ATP production, such mitochondrial dysfunction has been implicated in stress-related cognitive decline [48]. Mitochondrial ROS accumulation has also been reported to activate the JNK signaling pathway, accompanied by increased BAX phosphorylation and reduced expression of the anti-apoptotic protein BCl-2 [48,51]. This imbalance increases mitochondrial membrane permeability, facilitates cytochrome c release, and promotes downstream caspase activation [48,51]. Consequently, the intrinsic anti-apoptotic mechanism is weakened, accelerating neuronal damage [10,51]. These alterations, encompassing mitochondrial dysfunction and neuronal injury, have been reported to be closely associated with depression-related behavioral disturbances [23,50]. In alignment with these mechanisms, CUMS induced mitochondrial dysfunction and activation of JNK-dependent intrinsic apoptotic signaling (Figure 7). In contrast, ESP administration mitigated these pathological processes, thereby attenuating stress-induced neuronal dysfunction. These neuroprotective effects are likely driven by flavonoids contained in ESP. For example, kaempferol 3-O-glucoside alleviated metabolic disorders by suppressing mitochondrial structural damage and increasing ATP levels in a mouse model of LPS-induced depressive-like behavior [23]. Similarly, in an ischemia–reperfusion injury model, kaempferol 3-O-glucoside exhibited anti-apoptotic effects, including downregulation of BAX and caspase-3, and upregulation of BCl-2 expression [35]. Consistent with these findings, extracts from other Theaceae family plants, such as *Camellia japonica*, have also been reported to attenuate neuronal apoptosis by suppressing JNK phosphorylation in vivo [52]. This result suggests that ESP may mitigate mitochondrial dysfunction and apoptotic signaling under chronic stress conditions, supporting its potential neuroprotective effects in a preclinical stress model.

Prolonged exposure to stress induces neuronal dysfunction, including changes in synaptic plasticity, reduced neurogenesis, and dendritic atrophy, which are closely associated with cognitive impairment [53]. The hippocampus and prefrontal cortex are particularly vulnerable to stress, where neuronal loss and synaptic deficits are frequently observed [28]. These changes have been linked to stress-induced HPA axis hyperactivation and excessive glucocorticoid release, which have been shown to suppress BDNF expression [39,54]. Through TrkB-mediated CREB phosphorylation, BDNF promotes synapse-related gene expression and supports learning and memory [13,54]. Inhibition of the BDNF/TrkB pathway results in synaptic dysfunction and cognitive impairment [13]. Moreover, BDNF regulates ACh release, cholinergic neuronal maturation and survival, and AChE activity, and stress-induced reductions in BDNF may contribute to cholinergic dysfunction, thereby exacerbating cognitive impairment [14,55]. In parallel, inhibition of the BDNF/TrkB/CREB pathway can downregulate presynaptic and postsynaptic proteins, including SYP and PSD-95, which are essential for neurotransmitter release and synaptic stability [13,54]. This impairs synaptic function and ultimately exacerbates cognitive dysfunction [13,54]. In this study, CUMS exposure decreased ACh content and increased AChE activity, impairing cholinergic neurotransmission function and significantly reducing the expression levels of proteins related to synaptic plasticity in the cerebral cortex and hippocampus (Figure 8). In contrast, ESP administration effectively improved these changes, restoring cholinergic function and synaptic plasticity, and contributing to the alleviation of CUMS-induced cognitive impairment. In a Pb-induced cognitive dysfunction model, epicatechin was reported to improve cholinergic neurotransmission efficiency by inhibiting AChE activity and thereby reducing ACh degradation in the synaptic cleft [24]. Moreover, kaempferol 3-O-glucoside improved cognitive function by stabilizing the synaptic structure and promoting synaptic plasticity by increasing the expression of BDNF, SYP, and PSD-95 [23]. Furthermore, quercitrin was confirmed to promote neuroplasticity by activating the BDNF/CREB signaling pathway in the hippocampus of LPS-induced depressed mice [25]. Thus, ESP containing these various phenolic compounds may contribute to the improvement of CUMS-induced cognitive deficits through the recovery of cholinergic function and by enhancing synaptic plasticity in a preclinical model.

To further confirm these effects, we performed a correlation analysis that identified significant correlations between depression-related and cognitive dysfunction biomarkers (Figure 9). Elevated HPA-axis activity corresponded to increased inflammatory and apoptosis-related signals, together with diminished antioxidant defenses and synaptic function. In contrast, GR exhibited the opposite correlation pattern, which may reflect engagement of HPA negative feedback. Meanwhile, corticosterone showed an inverse correlation with serotonin and dopamine, indicating that stress-axis activation is accompanied by a reduction in monoamines. Taken together, these correlations suggest that HPA axis dysregulation is closely related to neuroinflammatory and apoptotic signaling and may be linked to synaptic dysfunction. These results provide a mechanistic insight into the cognitive and emotional impairments observed in this study. Nevertheless, several limitations should be acknowledged. The present study relied on the CUMS model in a single strain of male mice, which may not fully reflect the complexity and biological variability of stress-related neuropsychiatric disorders. In addition, although multiple stress-related pathways were examined, the causal interactions among these mechanisms were not directly tested and warrant further investigation. Another limitation relates to the extrapolation of dose to humans. Using the body surface area (BSA) conversion method recommended by the U.S. Food and Drug Administration (FDA) [56], the doses of 50 and 100 mg/kg used in this study correspond to estimated human equivalent doses (HEDs) of approximately 4 and 8 mg/kg, respectively. For a 60 kg adult, these values translate to approximately 240 and 480 mg/day. Although this conversion provides a preliminary reference for potential clinical investigation, these estimates remain theoretical and require validation in future clinical studies. Furthermore, a standard pharmacological comparator was not included as a positive control in the present study. In particular, no direct comparison was conducted with conventional antidepressants, such as fluoxetine or imipramine, which limits the clinical extrapolation of the present findings. Despite these limitations, the present findings provide meaningful insight into stress-related pathological mechanisms and suggest that ESP attenuates CUMS-induced depressive-like behaviors and cognitive dysfunction in a preclinical model through its antioxidant, anti-inflammatory, and neuroprotective effects.

## 4. Materials and Methods

### 4.1. Sample Preparation

*S. pseudocamellia* leaves utilized in this experiment were procured in dried form from Yagcho Maeul (Seongnam, Republic of Korea) in February 2024. The plant material was originally collected from Hongcheon, Gangwon Province, Republic of Korea (approximate latitude: 37.783560, longitude: 127.867707) in June 2023. The hot air-dried sample was subjected to extraction using 20% ethanol (50-fold, *w*/*v*) for 2 h at 40 °C, followed by filtration through No. 2 filter paper (Whatman PLC, Kent, UK). Following filtration, the extract was concentrated using a vacuum rotary evaporator (N-Nseries, Tokyo Rikakikai Co., Ltd., Tokyo, Japan). The resulting ESP was lyophilized and subsequently kept at −20 °C until each experimental use.

### 4.2. Identification and Quantification of Bioactive Compounds in ESP

#### 4.2.1. UPLC-Q-TOF-MS/MS System

The ESP was prepared by dissolving it in 50% methanol and used subsequently subjected for analysis. The bioactive compounds present in ESP were analyzed using a Nexera XS UPLC system (Shimadzu, Kyoto, Japan) connected to an X500R Q-TOF-MS (SCIEX, Framingham, MA, USA) equipped with an Acquity UPLC BEH C_18_ chromatographic column (2.1 mm × 100 mm, 1.7 μm; Waters, Milford, MA, USA) at the High-Tech Materials Analysis Core Facility of Gyeongsang National University (Jinju, Republic of Korea). The mobile phase comprised solvent A (distilled water containing 0.1% formic acid) and solvent B (acetonitrile containing 0.1% formic acid). The oven temperature was maintained at 40 °C, with a flow rate of 0.35 mL/min. The gradient elution conditions were as follows: 0–18 min solvent B: 0–80%; 18–20 min solvent B: 80–0%; 20–25 min solvent B: 0%. Following chromatographic separation, analytes were detected on a Q-TOF-MS functioning in the negative electrospray ionization (ESI^−^) mode. The MS conditions were as follows: lamp collision energy at 20–50 eV, ion source temperature at 500 °C, capillary voltage at 2.5 kV, and mass range from 50 to 1500 *m*/*z*. Data acquisition and compound identification were conducted utilizing SCIEX OS software (version 3.0.0.3339, SCIEX, Framingham, MA, USA).

#### 4.2.2. Quantitative Determination Using HPLC-DAD

Quantitative analysis of bioactive compounds was performed using HPLC-DAD (Ultimate 3000 series, Dionex, Sunnyvale, CA, USA) according to a previously reported method [18]. Chromatographic separation was achieved on a YMC-Triart C_18_ column (250 × 4.6 mm, 5 μm; YMC Korea, Seongnam, Republic of Korea). The mobile phase comprised solvent A (distilled water containing 0.1% formic acid) and solvent B (acetonitrile containing 0.1% formic acid) at a flow rate of 1.0 mL/min. The gradient elution conditions were as follows: 0–15 min solvent B: 0–30%; 15–30 min solvent B: 30–60%; 30–40 min solvent B: 60–100%; 40–42 min solvent B: 100–0%. Chromatograms were recorded at 260 nm. Quercitrin and rutin were selected as major marker compounds. Quercitrin was quantitatively analyzed in the present study, whereas the rutin content had been previously determined in our study [18]. For quantification, standard solutions of quercitrin were prepared, diluted in series, and analyzed under identical chromatographic conditions. A calibration curve was constructed using peak areas, and the quercitrin content in the extract was calculated.

### 4.3. Animal Design

**Figure 10:**
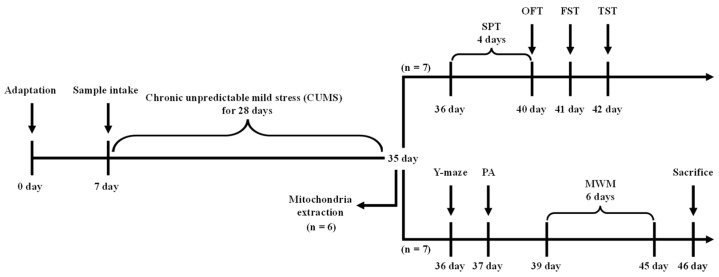
Experimental design for assessment of depressive-like behaviors and cognitive dysfunction in chronic unpredictable mild stress (CUMS)-induced mice.

C57BL/6 male mice aged 4 weeks were supplied by Samtako (Osan, Republic of Korea). To maintain objectivity, the mice were randomly allocated to experimental groups using the RANDARRAY and SORTBY functions in Microsoft Excel (version 365, Microsoft Corp., Redmond, WA, USA). Mice were housed five per cage and maintained under standard laboratory conditions at a constant temperature (22 ± 2 °C), relative humidity (approximately 55%), and a 12-h light/dark cycle. To mitigate potential biases, the sequences of treatments and cage arrangements were randomly assigned and rotated regularly. There were six groups based on the type of treatment that consisted of the NC group (non-CUMS treatment + drinking water), normal sample (NS) group (non-CUMS treatment + ESP 100 mg/kg of body weight), CUMS group (CUMS treatment + drinking water), Thea group (CUMS treatment + L-theanine 4 mg/kg of body weight) as positive control, ESP 50 group (CUMS treatment + ESP 50 mg/kg of body weight), and ESP 100 group (CUMS treatment + ESP 100 mg/kg of body weight). The dose of ESP was determined based on previous studies that established the biologically effective range of the Theaceae extract [32,57]. Each experimental group consisted of 20 mice, which were pre-assigned to independent analyses before study initiation to ensure statistical reliability while minimizing animal use. Specifically, mice were allocated for behavioral tests (*n* = 7), biochemical assays of the antioxidant and cholinergic systems (*n* = 5), analysis of hormonal and mitochondrial function (*n* = 5), and protein expression analysis via Western blotting (*n* = 3). The samples were dissolved in drinking water and administered orally to the mice once daily for 4 weeks via gavage. All animal experimental protocols were reviewed and approved by the Institutional Animal Care and Use Committee (IACUC) of Gyeongsang National University (GNU-241107-M0213, date of approval: 7 November 2024) and were conducted in compliance with institutional animal care guidelines. A schematic of the experimental design is presented in Figure 10. Mice were subjected to CO_2_ euthanasia after the completion of behavioral tests.

### 4.4. CUMS Procedures

After a one-week adaptation period, mice received daily exposure to one of seven randomly assigned stressors for 4 weeks along with sample administration. The stressors included food or water deprivation (24 h), cage tilting (24 h), wet bedding (24 h), cage swap (24 h), empty cage exposure (24 h), nocturnal light exposure (12 h), and mild restraint (2 h), with detailed induction procedures provided in Table 3.

### 4.5. Behavioral Tests

#### 4.5.1. SPT

The SPT was performed to assess anhedonia-like behavior, characterized by a reduced response to rewarding stimuli. The experiment was conducted for 4 days. Initially, each mouse was housed individually in a separate cage and provided with two bottles filled with a 1% sucrose solution for 24 h. On the following day, one of the sucrose solution bottles was substituted with drinking water, and after 12 h, the bottles were repositioned to prevent a preference for a specific position. On the third day, water and food in the cage were removed for 24 h. On the last day, the consumption of each bottle was measured. The sucrose preference was calculated as a percentage of 1% sucrose solution consumption in terms of the total amount of consumption.

#### 4.5.2. OFT

The OFT was conducted in a white acrylic chamber (50 × 50 × 50 cm) to assess spontaneous locomotor activity and anxiety-related behaviors. The field was divided into a center zone (25 cm × 25 cm) and the rest of the peripheral zone. Each mouse was positioned in the peripheral zone and permitted to explore the field freely for 5 min. The time spent in the center zone was recorded using a video tracking system (Smart 3.0, Panlab, Barcelona, Spain).

#### 4.5.3. FST

The FST was conducted to evaluate behavioral despair under inescapable stress. Mice were placed in an open cylinder acrylic container (10 cm × 50 cm) filled with 15 cm of water maintained at 25 ± 1 °C. The immobility time was recorded for 5 min using a video tracking system (Smart 3.0, Panlab).

#### 4.5.4. TST

The TST was conducted to examine behaviors indicative of helplessness by measuring the duration of immobility exhibited in response to short-term inescapable stress. Mice were suspended by the tail with medical adhesive tape affixed approximately 1 cm from the tip and looped over a horizontal iron rod positioned on top of a white acrylic box (50 cm × 50 cm × 50 cm). Immobility time was recorded for 5 min using a video tracking system (Smart 3.0, Panlab).

#### 4.5.5. Y-Maze Test

The Y-maze test was performed to evaluate spatial working memory through the analysis of spontaneous alternation behavior. The Y-maze apparatus comprised three white plastic arms, and the experimental procedure commenced upon positioning the mouse at the terminus of a specified arm. The number of entries into each arm and crossing behaviors were recorded for 8 min using a video tracking system (Smart 3.0, Panlab).

#### 4.5.6. PA Test

The PA test was conducted to assess both short- and long-term memory following exposure to an aversive stimulus. The PA apparatus consisted of two compartments, one bright and one dark, which were interconnected by a passage. On the first day, mice were positioned within the bright compartment with the lights turned off and allowed to acclimate for 1 min. Subsequently, the lights were turned on, and the mice remained in the same compartment for 2 min to adapt to the illuminated environment. Then, Upon the opening of the central separation door, and after the mouse had fully entered the dark compartment with all four paws, a 0.5 mA foot shock was delivered for 3 s. After 24 h, under conditions identical to those of the initial day, the latency to enter the dark compartment from the light compartment was measured up to 300 s.

#### 4.5.7. MWM Test

The MWM test was performed to evaluate spatial learning and long-term memory. The experimental setup featured a circular pool (90 cm × 30 cm) partitioned into four equal zones designated as north (N), south (S), east (E), and west (W). The pool was contained with water mixed with non-toxic white ink and maintained at a temperature of 25 ± 1 °C. Visual cues were positioned in each quadrant, and the escape platform located at the center of the W zone. On the first day, the escape platform was placed 1 cm above the water surface, and the mice were permitted to swim for 1 min to facilitate learning of the platform location. From the second to the fourth day, the escape platform was submerged 1 cm below the surface, and the latency to locate the platform was recorded within 1 min. Finally, on the sixth day, the escape platform was taken out of the pool, and the retention time in the W zone was recorded for 1 min using a video tracking system (Smart 3.0, Panlab).

### 4.6. Hormonal Analysis

To quantify hormone levels, blood was obtained from the abdominal vein of mice after behavioral testing. The serum was isolated through the centrifuging whole blood at 10,000× *g* for 15 min at 4 °C and kept at −80 °C until further analysis. The serum levels of corticosterone, dopamine, and serotonin were quantified using enzyme-linked immunosorbent assay (ELISA) kits (MyBioSource, San Diego, CA, USA) in line with the instructions provided by the manufacturer.

### 4.7. Antioxidant System

#### 4.7.1. GSH Level

To measure GSH level, brain tissues obtained from mice were homogenized in 10 mM phosphate buffer (pH 6.0) containing 1 mM ethylenediaminetetraacetic acid. The homogenates were subsequently centrifuged at 10,000× *g* for 15 min at 4 °C. The supernatant was combined with 5% metaphosphoric acid in a 1:1 (*v*/*v*) ratio and centrifuged again at 2000× *g* for 2 min at 4 °C. Subsequently, the supernatant was mixed with 0.26 M Tris-HCl buffer (pH 7.5), 0.65 N sodium hydroxide (NaOH), and 1 mg/mL *o*-phthalaldehyde in methanol. The mixture was incubated in the dark for 15 min, and the fluorescence intensity measurements were performed using a fluorometer (Infinite F200, Tecan, Mannedorf, Switzerland), with excitation and emission wavelengths configured at 360 and 430 nm, respectively.

#### 4.7.2. SOD Level

To assess SOD level, brain tissues collected from mice were homogenized in phosphate buffered saline (PBS, pH 7.4) and then centrifuged at 400× *g* for 10 min at 4 °C. The obtained pellet was mixed with 1× cell extraction buffer containing 10× SOD buffer, 20% Triton X-100, distilled water, and 200 mM phenylmethane sulfonyl fluoride in 95% ethanol. The mixture was reacted on ice for 30 min with vortexing performed at 5 min intervals. Afterward, the reactant was centrifuged at 10,000× *g* for 10 min at 4 °C, and the supernatant was analyzed using a SOD analysis kit (Dojindo Molecular Technologies, Rockville, MD, USA).

#### 4.7.3. MDA Content

To evaluate MDA content, brain tissues obtained from mice were homogenized in PBS (pH 7.4) and centrifuged at 2356× *g* for 10 min at 4 °C. The resulting supernatant was combined with 1% phosphoric acid and 0.67% thiobarbituric acid, followed by incubation in a water bath maintained at 95 °C for 1 h. After incubation, the reactant was cooled by centrifuging at 5000× *g* for 1 min at 4 °C, and the supernatant was analyzed using a spectrophotometer (UV-1800, Shimadzu, Tokyo, Japan) configured at 532 nm.

### 4.8. Mitochondrial Function

#### 4.8.1. Extraction of Mitochondria from Brain Tissues

To assess mitochondrial function, mitochondria were isolated from brain tissues following a previously described protocol [58].

#### 4.8.2. ROS Level

To measure mitochondrial ROS level, the mitochondrial extract from brain tissues was combined with 2′,7′-dichlorofluorescin diacetate solution prepared in respiration buffer (pH 7.0). The respiration buffer containing 125 mM KCl, 2 mM KH_2_PO_4_, 2.5 mM malate, 20 mM HEPES, 1 mM MgCl_2_, 5 mM pyruvate, and 500 μM egtazic acid. The reactant was incubated for 20 min under dark conditions, and the fluorescence intensity measurements were performed using a fluorometer (Infinite F200, Tecan), with excitation and emission wavelengths configured at 485 and 535 nm, respectively.

#### 4.8.3. MMP

To evaluate MMP, the mitochondrial extract from brain tissues was mixed with 1 μM 5,5′,6,6′-Tetrachloro-1,1′,3,3′-tetraethylbenzimidazolylcarbocyanine iodide (JC-1) solution prepared in assay buffer containing 5 mM pyruvate and 5 mM malate. The reactant was incubated for 20 min under dark conditions, and the fluorescence intensity measurements were performed using a fluorometer (Infinite F200, Tecan), with excitation and emission wavelengths configured at 535 and 590 nm, respectively.

#### 4.8.4. ATP Content

To assess mitochondrial ATP content, the mitochondrial extract from brain tissues was centrifuged at 13,000× *g* for 10 min at 4 °C. The resulting pellet was resuspended in 25 mM Tris-acetate buffer (pH 7.75) containing 1% trichloroacetic acid and centrifuged at 10,000× *g* for 15 min at 4 °C. The mitochondrial ATP content in the supernatant was quantified by a luminometer (Glomax^®^, Promega Corporation, Madison, WI, USA) utilizing an ATP kit (Promega Corporation, Madison, WI, USA).

### 4.9. Cholinergic System

#### 4.9.1. ACh Content

To measure ACh content, brain tissues collected from mice were homogenized in PBS (pH 7.4) and centrifuged at 13,572× *g* for 30 min at 4 °C. The supernatant was mixed with an alkaline hydroxylamine reagent containing 2 M hydroxylamine dissolved in 1 N hydrochloric acid (HCl) and 3.5 N NaOH at a 1:1 ratio for 1 min. Subsequently, 0.5 N HCl and 0.37 M FeCl_3_ · 6H_2_O in 0.1 N HCl were incorporated, and the ACh content was analyzed using a microplate reader (Epoch2, BioTek Instruments Inc., Winooski, VT, USA) configured at 540 nm.

#### 4.9.2. AChE Activity

To measure AChE activity, brain tissues collected from mice were homogenized in PBS (pH 7.4) and centrifuged under the same conditions as the ACh content assay. The supernatant was mixed with 50 mM sodium phosphate buffer (pH 7.4) and incubated for 15 min at 37 °C. After that, a substrate solution (0.5 mM acetylthiocholine iodide and 1 mM 5,5′-dithiobis (2-nitrobenzoic acid)) was added and incubated for 10 min at 37 °C. The AChE activity was analyzed using a microplate reader (Epoch2, BioTek Instruments Inc.) configured at 405 nm.

### 4.10. Western Blot Analysis

The Western blot analysis of whole brain, cerebral cortex, and hippocampus tissues was carried out according to a previously reported procedure [58]. Table 4 presents detailed information regarding the primary and secondary antibodies employed in this study.

### 4.11. Statistical Analysis

All results are expressed as the mean ± standard deviation (SD). The normality of the data distribution and the homogeneity of variances were evaluated using the Shapiro–Wilk and Brown–Forsythe tests, respectively. When the data satisfied both assumptions, one-way analysis of variance (ANOVA) followed by Tukey’s post hoc test was performed. If not, the Kruskal–Wallis test was conducted, followed by Dunn’s post hoc analysis. Statistical analyses were conducted utilizing GraphPad Prism 10 (GraphPad Software, Boston, MA, USA). A *p*-value of less than 0.05 was regarded as indicative of statistical significance. Pearson correlation coefficients between behavioral tests and key mechanistic biomarkers were calculated in R (version 4.5.1, R Foundation for Statistical Computing, Vienna, Austria) and visualized as a heatmap.

## 5. Conclusions

This study evaluated the stress-attenuating effects of ESP and its impact on cognitive function in a CUMS-induced chronic stress model. Our results indicate that ESP significantly improved abnormalities related to depression- and anxiety-like behaviors. Similarly, ESP enhanced cognitive function by restoring spatial memory and learning ability. Additionally, ESP restored hormonal balance by regulating CUMS-induced HPA axis hyperactivation and strengthening the antioxidant defense system, contributing to nervous system homeostasis. Furthermore, ESP alleviated neuroinflammatory responses by inhibiting the TLR4/NF-κB pathway and suppressed apoptosis by preserving mitochondrial function and regulating the JNK pathway. Moreover, ESP promoted neuroplasticity and synaptic function by enhancing BDNF/TrkB signaling and increasing the expression of proteins related to synapses. Such protective effects of ESP are presumed to be attributable to the actions of plant-derived bioactive compounds, such as quercitrin, rutin, and kaempferol 3-O-glucoside. In conclusion, the findings of this study suggest that ESP mitigates depressive-like behaviors and cognitive dysfunction in a preclinical model through modulation of hormonal imbalance, oxidative stress, inflammation, and synaptic dysfunction.

## Figures and Tables

**Table 1:** Bioactive compounds identified in 20% ethanolic extract from *Stewartia pseudocamellia* Maxim. leaves (ESP) using UPLC-Q-TOF-MS/MS analysis.

No.	Retention Time (Min)	Compound	Parent Ion (*m*/*z*)	Fragment Ion (*m*/*z*)
1	3.602	Procyanidin B2	577	425, 407, 289
2	3.991	Epicatechin	579	289, 245
3	5.773	Rutin	609	301, 300, 271, 255, 151
4	5.845	Catechin gallate	441	289, 169, 125
5	6.174	Kaempferol 3-O-glucoside	447	285, 284, 255, 227
6	6.424	Quercitrin	447	301, 300, 151

**Table 2:** Effect of 20% ethanolic extract from *Stewartia pseudocamellia* Maxim. Leaves (ESP) on stress-related hormones change in serum in CUMS-induced mice.

			Unit: ng/mL
	NC	CUMS	ESP100
Corticosterone	198.00 ± 14.83 ^c^	334.00 ± 11.40 ^a^	238.00 ± 14.83 ^b^
Dopamine	60.72 ± 4.20 ^a^	39.65 ± 3.95 ^b^	56.16 ± 4.44 ^a^
Serotonin	54.30 ± 4.53 ^a^	42.16 ± 4.31 ^b^	52.45 ± 6.53 ^a^

Values are presented as the mean ± SD (*n* = 5). Different lowercase letters (a–c) indicate statistically significant differences between groups (*p* < 0.05). Means marked with the same letter indicate no statistically significant differences between groups. One-way ANOVA followed by Tukey’s post hoc test was applied for statistical analysis.

**Table 3:** Stressors applied to induce chronic unpredictable mild stress (CUMS) in mice.

Stressors	Time	Description
Food or water deprivation	24 h	Access to food or water was temporarily withheld from the mice.
Cage tilting	24 h	Each cage housing the mice was tilted to approximately a 45° angle.
Wet bedding	24 h	A wet bedding condition was produced by adding 200 mL of distilled water to the cages housing the mice.
Cage swap	24 h	Mice were transferred from their home cages to unfamiliar cages previously occupied by other mice.
Empty cage	24 h	Mice were housed in empty cages without bedding.
Nocturnal light exposure	12 h	Continuous light was applied overnight to the mice.
Mild restraint (via placement in small cages)	2 h	Mice were placed in small plastic cages with ventilation holes on every side except the top and bottom.

**Table 4:** A detailed list of the primary and secondary antibodies used in this experiment.

Antibody	Catalog No.	Manufacturer
Anti-mouse IgG	AP124P	Millipore (Billerica, MA, USA)
Anti-rabbit IgG	#7074	Cell Signaling Tech (Danvers, MA, USA)
BDNF	#47808	
Caspase-3	#9662	
p-CREB-1	#9198	
ChAT	20747-1-AP	Proteintech (Rosemont, IL, USA)
AChE	sc-373901	Santa Cruz Biotech (Dallas, TX, USA)
ACTH	sc-57018	
β-actin	sc-69879	
BCl-2	sc-7382	
BAX	sc-7480	
Caspase-1	sc-392736	
CRF	sc-293187	
CYP11B1	sc-374096	
GR	sc-393232	
HO-1	sc-136960	
IL-1β	sc-515598	
Keap1	sc-514914	
MyD88	sc-74532	
Nrf2	sc-365949	
p-JNK	sc-6254	
p-NF-κB	sc-136548	
p-IκB-α	sc-8404	
PSD-95	sc-32290	
SYP	sc-17750	
TLR4	sc-52962	
TrkB	sc-377218	
TNF-α	sc-33639	

## Data Availability

The original contributions presented in this study are included in the article/Supplementary Material. Further inquiries can be directed to the corresponding author.

## Supplementary Materials

The following supporting information can be downloaded at: https://www.mdpi.com/article/10.3390/ph19030354/s1, Figure S1: Effect of 20% ethanolic extract from *Stewartia pseudocamellia* leaves (ESP) in corticosterone-induced cytotoxicity in HT22 and MC-IXC cells.
