# Supplementary material for: Antidepressant and Cognitive-Enhancing Effects of Stewartia pseudocamellia Maxim. Leaves in Chronic Unpredictable Mild Stress-Induced Mice Through HPA Axis Regulation and the BDNF/TrkB Pathway
Source: Pharmaceuticals (Basel). 2026 Feb 25;19(3):354. doi: 10.3390/ph19030354 (PMC13028786; doi:10.3390/ph19030354)
Supplement: Supplementary file 1 [file pharmaceuticals-19-00354-s001.zip › pharmaceuticals-4136823-supplementary.pdf]

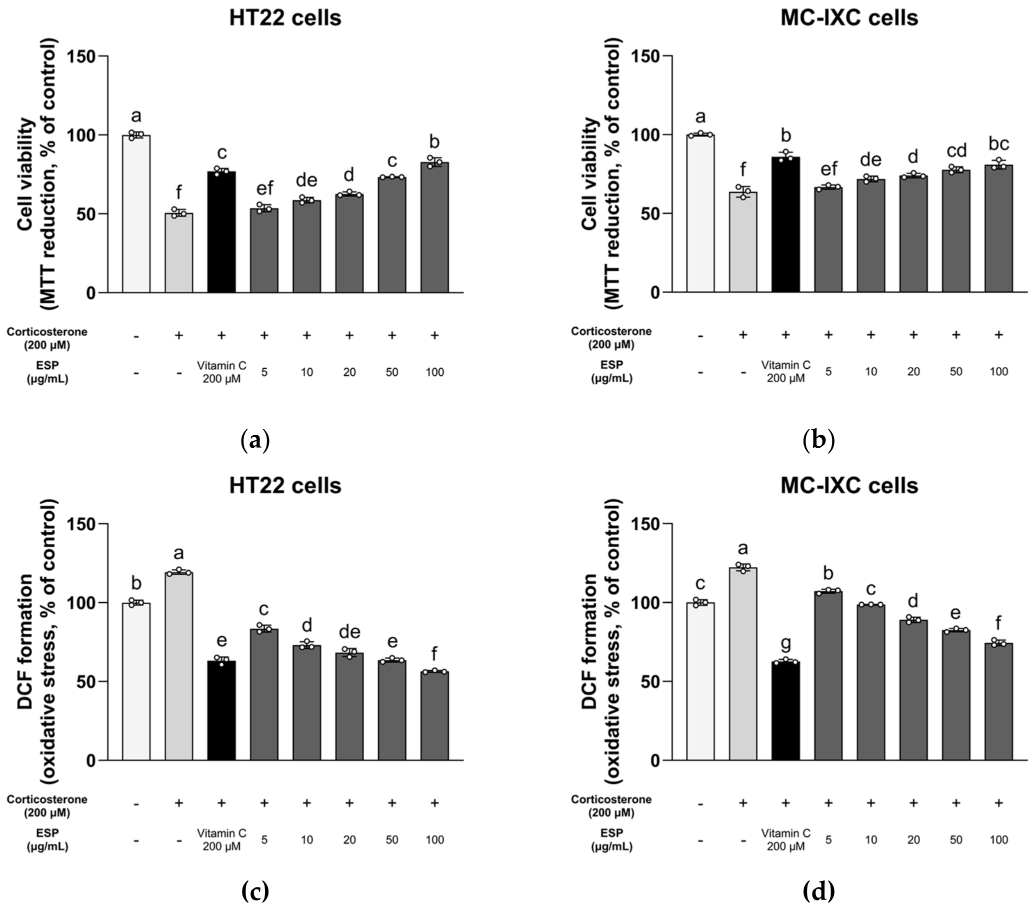

Figure S1. Effect of 20% ethanolic extract from *Stewartia pseudocamellia* Maxim. leaves (ESP) in corticosteron e-induced cytotoxicity in HT22 and MC-IXC cells. Cell viability (a and b) and intracellular reactive oxygen species (ROS) production (c and d). Values are presented as mean ± SD (n = 3). Different lowercase letters (a–g) above the bars indicate statistically significant differences between groups (p < 0.05). Bars marked wi th the same letter indicate no statistically significant differences between groups. One-way ANOVA followe d by Tukey's post hoc test was applied for statistical analysis.
